# Case report: A rare case of urinary myiasis induced by the fourth instar larvae of *Telmatoscopus albipunctatus*

**DOI:** 10.1371/journal.pntd.0006016

**Published:** 2017-12-07

**Authors:** Beibei Zhang, Lifu Wang, Jiahua Liu, Lian Xu, Langui Song, Xiaoying Wu, Xi Sun, Zhongdao Wu

**Affiliations:** 1 Department of Parasitology of Zhongshan School of Medicine, Sun Yat-sen University, Guangzhou, Guangdong, China; 2 Key Laboratory of Tropical Disease Control (SYSU), Ministry of Education, Guangzhou, Guangdong, China; 3 Provincial Engineering Technology Research Center for Biological Vector Control, Guangzhou, Guangdong, China; 4 School of Public Health, Fudan University, Shanghai, China; National Institute of Allergy and Infectious Diseases, UNITED STATES

## Abstract

*Telmatoscopus albipunctatus*, a cosmopolitan fly, is widely distributed throughout moist environments. It is one of the most medically important insects (especially in urban environments) that may potentially cause myiasis. Urinary myiasis and other sites of infestation, including the intestine, nasal passages, lung, and derma, have been reported. This is the first case report of a Chinese middlescent woman infected with *T*. *albipunctatus* in Guangzhou, China. In the present report, a 50-year-old woman came to The Third Affiliated Hospital of Southern Medical University, Guangzhou, China, because larvae were found when urinating in the morning; this had occurred every two days within the past two months. She complained of frequent micturition and urgency. Urine tests indicated that all indexes were normal except for slight urinary tract infection. Subsequently, the larvae were sent to the diagnostic section for parasitic infection in the Department of Parasitology, Zhongshan School of Medicine, Sun Yat-sen University, Guangzhou, China. The stereoscopic microscope and transmission electron microscope were used for morphological observation. On this basis, the cytochrome oxidase subunit 1 (*COX1*) gene was specifically amplified by PCR. Sequence analysis of the PCR product and phylogenetic analysis were used to identify the species. Morphological analysis combined with molecular biology methods indicated that the insect was the fourth instar larvae of *T*. *albipunctatus*. Our results show that this was a case of a 50-year-old woman infected with *T*. *albipunctatus* larvae in her urinary tract, and the findings suggest that clinicians should be vigilant for this infection.

## Introduction

*Psychodidae* is a cosmopolitan fly that is tiny and hairy. It belongs to the family Nematoceran and is a medically important insect, especially in urban areas. Most of the adults are distributed throughout houses, stinking ditches, or septic tanks, and the larvae are bred in moist environments such as toilets and bathrooms [1]. Because of their unique lifestyle, many sewage microorganisms and viruses may be carried indoors, introducing potentially unhealthy situations [2]. Myiasis is the most common disease resulting from the accidental infestation of living humans or other animals with these fly larvae [3]. Urethral myiasis and other sites of infestation (including intestine, nasal passages, and lungs) have been reported elsewhere [3–6].

*T*. *albipunctatus* is one member of Psychodidae. Because several conventional control measures such as the use of chemical insecticides have been used to kill these nuisance moth flies, little has been reported regarding this type of case [7][8]. The latest case report concerns a 21-year-old male patient in Taiwan who experienced intestinal myiasis caused by larvae of *T*. *albipunctatus* [1]. Therefore, many clinicians lack the ability to identify the insect accurately, which may lead to misdiagnosis and mistreatment and cause non-negligible suffering of the patient [9].

In this report, we first present a 50-year-old woman in Guangzhou, China, who was infected with the fourth instar larvae of *T*. *albipunctatus* in her urethra. The patient was diagnosed by a morphological examination combined with molecular biological techniques, which provided a more sensitive diagnosis method and timely, effective treatment for the disease.

## Case presentation

A 50-year-old woman who lives in a small town came to The Third Affiliated Hospital of Southern Medical University in Guangzhou, China, because of a urinary tract infection she had had for two months. She complained of frequent micturition and urgency. In addition, three to five larvae had been found in her morning urine, which had occurred every two days. According to the urine tests, all indexes were normal but accompanied by a slight urinary tract infection. Because this case was rare in the clinical context, it was difficult for the urologist to identify the larvae. To make an exact diagnosis, a morning urine sample was collected in a clean specimen container following the guidance of a nurse in the hospital and subsequently sent to the diagnostic section in the Department of Parasitology of the Sun Yat-sen University Zhongshan School of Medicine in Guangzhou, China.

**Fig 1:**
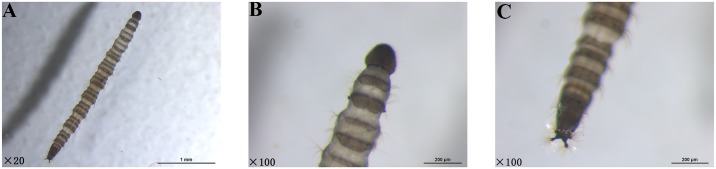
Morphologic examination of *T*. *albipunctatus* larvae under stereoscopic microscope. (A) View of the whole worm. (B) View of the head. (C) View of the tail.

**Fig 2:**
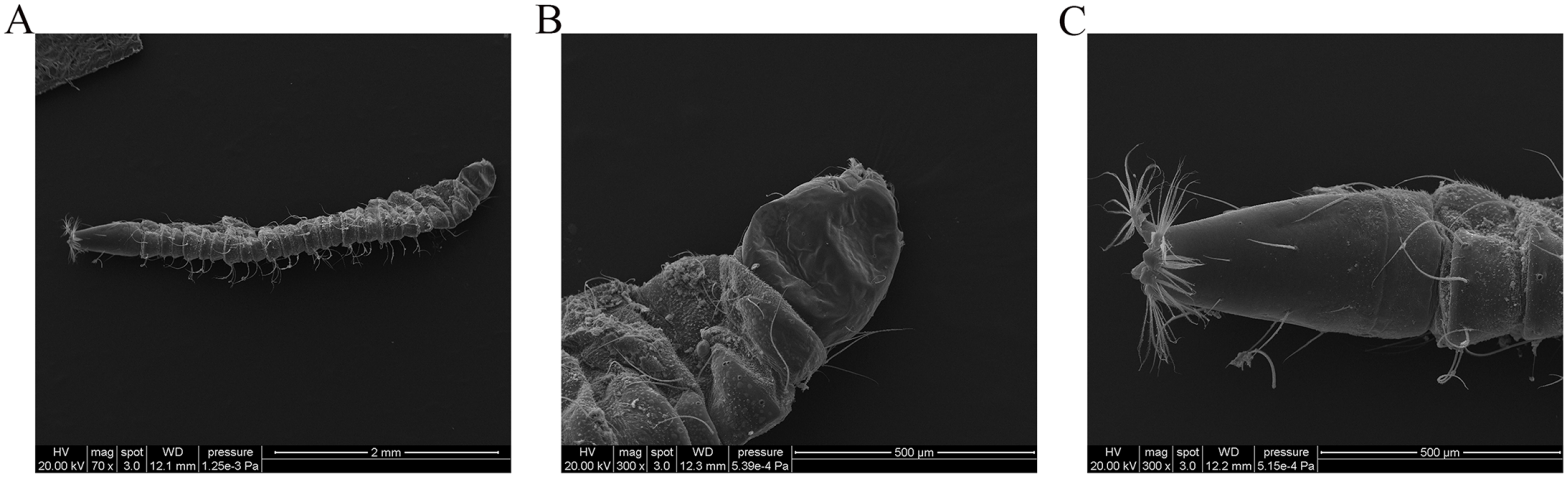
Morphologic examination of *T*. *albipunctatus* larvae by transmission electron microscope. (A) View of the whole worm. (B) View of the head. (C) View of the tail. HV, high vacuum; WD, working distance.

The larvae were dark brown, slender, and three to four millimeters long by gross observation. In addition, the two terminals were thinner than the body. Subsequently, a stereoscopic microscope and transmission electron microscope were used for further identification. As shown in Figs 1 and 2, the head of the larva was cone shaped and had chewing mouthparts. There were several protuberances on the tails, and radial long hairs extended from each protuberance. Based on this morphological observation, we presumed these larvae were fourth instar larvae and may be a species of Nematoceran.

**Fig 3:**
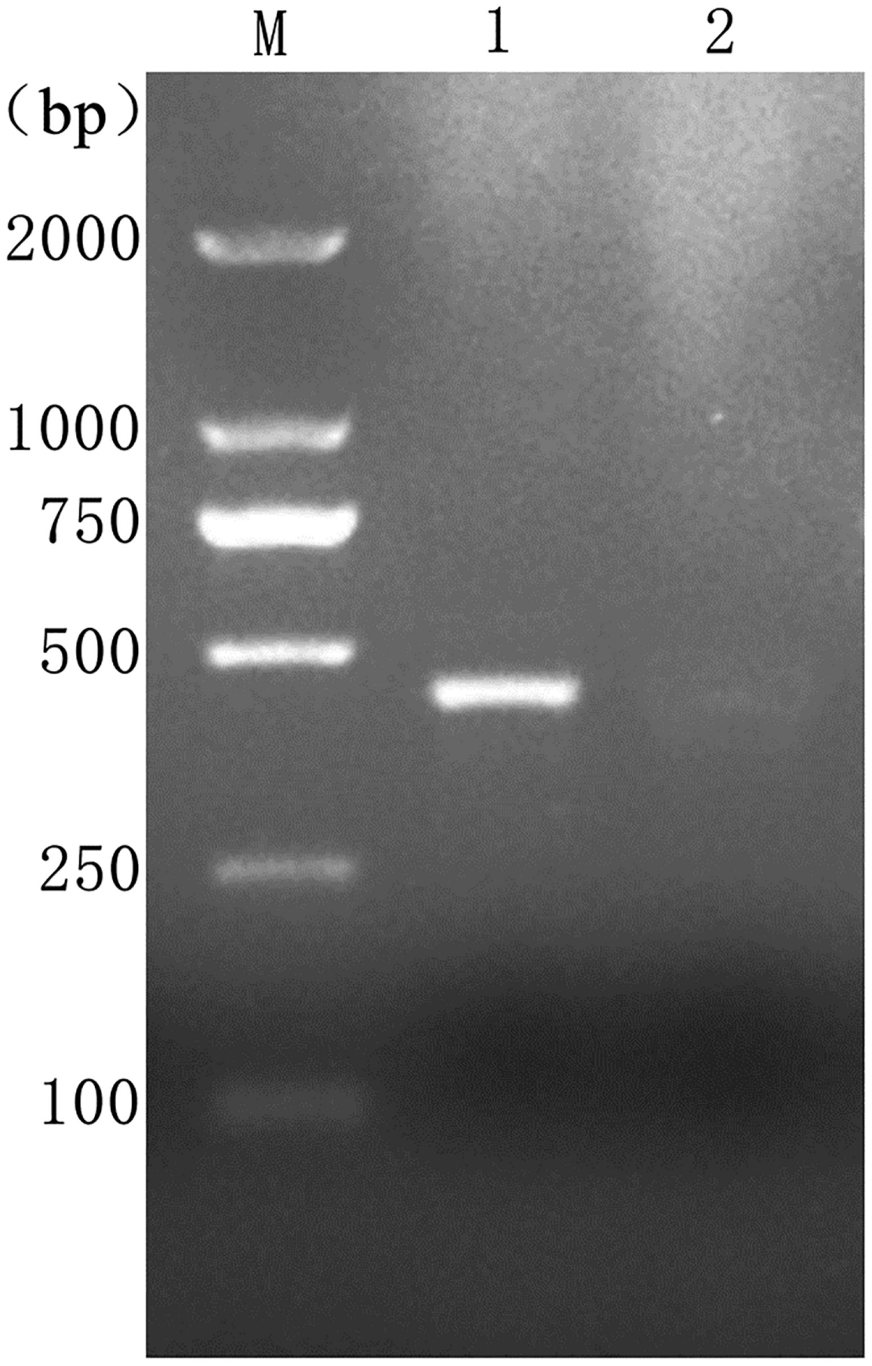
Representative electrophoresis result of *COX1* gene. Lane M: DL2000 DNA marker; Lanes 1: *COX1* PCR product; Lanes 2: PCR product without DNA. bp, base-pairs; COX1, cytochrome oxidase subunit 1; DL, DNA Ladder; M, Marker.

The next step was to clarify the larval species. All the procedures were followed as previously described [10]. Briefly, the HiPure Tissue DNA Mini Kit (Magen, China) was used to extract the genomic DNA from the larvae. The following protocol was conducted to amplify these genes: pre-denaturation at 95°C for 5 min, 35 cycles at 95°C for 50 s, 52 to 60°C for 50 s, and 72°C for 50 s, with a final extension at 70°C for 10 min. The DNA products were subsequently used for gel electrophoresis. The primer sequences were the following: forward primer: 5′-TGTATCCCACGCCGGAGCTTCA-3′; reverse primer: 5′ TCACCTCCTCCTGCTGGGTCAA -3′. Fig 3 shows the result of the PCR analyses. These results indicated that the larvae were *Psychodidae*.

To confirm the PCR result, genetic sequencing was performed to clarify the larval species. The purified cytochrome oxidase subunit 1 (*COX1*) gene product was sequenced by the company Majorbio. Finally, after homology searching with the Nucleotide BLAST in the National Center for Biotechnology Information (NCBI) database (http://blast.ncbi.nlm.nih.gov/Blast.cgi), the sequence exhibited 96% homology with *T*. *albipunctatus COX1* gene (Accession No. AB907184.1). The alignments displayed the following: score = 760 bits (411), expect = 0.0, identities = 415/417 (99%), gaps = 0/417 (0%), and strand = Plus/Plus. All these data, ultimately, finally indicated that the larvae were fourth instar larvae of *T*. *albipunctatus*.

**Fig 4:**
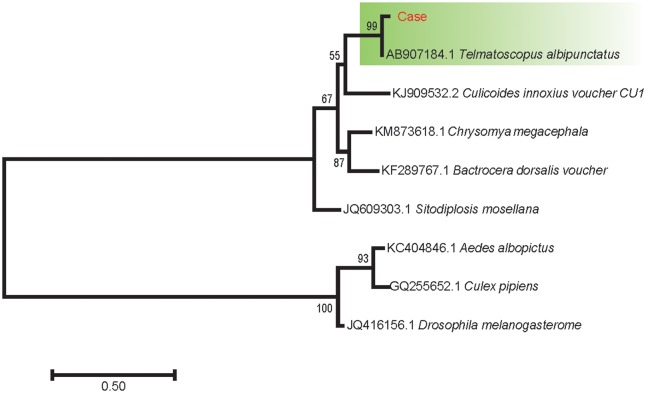
Phylogenetic analysis of *COX1* gene. The Maximum Likelihood phylogenic tree of *COX1* in the sequence of case product and related species using MEGA7.0.14 and edited in Figtree v1.4.2. Multiple sequence alignment was performed by CLUSATLW with default. COX1, cytochrome oxidase subunit 1.

Finally, the obtained sequence of *COX1* was aligned with a reference sequence downloaded from GenBank (https://www.ncbi.nlm.nih.gov/nuccore/AB907184.1). Phylogenetic analysis was used by comparing of sequences that were downloaded from GenBank (AB907184.1, KJ909532.2, KM873618.1, KF289767.1, JQ609303.1, KC404846.1, GQ255652.1, and JQ416156.1; Table 1). CLUSATLW with default parameters was used for multiple sequence alignment. Then, the phylogenetic tree was reconstructed with MEGA7.0.14 using a maximum likelihood method analysis and edited with Figtree v1.4.2. The robustness of clusters was assessed by bootstrap values using 1,000 replicates. As the results show in Fig 4, the sequence in this case was clustered closer to the *T*. *albipunctatus* reference sequence (Fig 4).

**Table 1:** Accession numbers of genes.

Name	Accession numbers	Database
*Telmatoscopus albipunctatus*	AB907184.1	NCBI
*Culicoides innoxius voucher CU1*	KJ909532.2	NCBI
*Chrysomya megacephala*	KM873618.1	NCBI
*Bactrocera dorsalis voucher*	KF289767.1	NCBI
*Sitodiplosis mosellana*	JQ609303.1	NCBI
*Aedes albopictus*	KC404846.1	NCBI
*Culex pipiens*	GQ255652.1	NCBI
*Drosophila melanogasterome*	JQ416156.1	NCBI

Abbreviation: NCBI, National Center for Biotechnology Information.

Based on our diagnosis, the patient was treated with broad-spectrum antibiotics for one week, which significantly alleviated the symptoms of frequent micturition and urgency. No larvae could be found in the urine at her last visit. In summary, all the results proved that the larvae in this case were *T*. *albipunctatus*.

## Discussion

*T*. *albipunctatus* is a primitive dipteran of the family Psychodidae whose breeding environment is partial to rotten and wet vegetation. The larvae of *T*. *albipunctatus* are commonly found in sewage, bathrooms, and toilets [1]. Although it is relatively rare for humans to be infected with such an insect, it still remains frequent in tropical countries, especially in rural areas with poor sanitation. Once the larvae opportunistically enter body cavities such as the nasopharyngeal passage, intestine, and urinary tract, these organs may create a breeding ground for infection, including myiasis. In the present case, we confirmed that a 50-year-old woman contracted urinary myiasis caused by *T*. *albipunctatus* infection. It has been previously reported that urinary myiasis may be associated with urinary tract pathology [11]. This urinary tract pathology is induced by inflammatory toxins, microorganisms, and viruses secreted by the larvae, and the larval migration can result in progressive and continuous necrosis of bladder wall [12]. In addition to *T*. *albipunctatus*, several urogenital myiasis cases caused by other species were reported in the past. For example, urogenital myiasis caused by *Psychoda albipennis* was reported in a 10-year-old girl, and *Eristalis tenax* was reported in a 58-year-old woman. Such patients may complain of urinary frequency, irritation, dysuria, and itching. Vomiting and side pain may also be observed [13][14].

All these cases indicated that poor sanitation and unhygienic domestic environments, including adverse living conditions, overcrowding, poor ventilation, and inadequate sewage systems, create higher risks for urinary myiasis [15]. Thus, environmental health approaches should be the primary approach to controlling urogenital myiasis. Poor personal hygiene, individual health conditions (immunosuppressed or immunocompromised status), low mobility, and ulcerating lesions are other risk factors for urinary myiasis and should be considered [3]. In this case, we speculated that the patient was infected by the larvae by urinating into unsanitary toilets or sleeping at night in warm weather without a covering. The anatomical and physiological characteristics of the female urethra and poor personal hygiene may have increased her likelihood of infection. Fortunately, when the patients improve their hygienic consciousness and sanitary conditions, they could recover without any symptoms after being treated with antibiotics and urinary tract antiseptic drugs.

Because of the conventional control measures used to kill *T*. *albipunctatus*, little has been reported regarding cases with infection by this insect, and many clinicians lack the ability to accurately identify these insects. In addition, the morphology of this larva causing urinary myiasis is similar to that of other insects such as Muscidae, Phlebotomidae, Culicoides, and Culicidae. Thus, the diagnosis of *T*. *albipunctatus* remains particularly low in sensitivity, and the delay in suboptimal antiparasitic care is common [7][8]. Therefore, more sensitive detection methods should be developed. In this case, we speculated that it was the fourth instar larvae of *Psychodidae* by stereoscopic microscope and transmission electron microscope. Molecular biological methods of sequence analysis and phylogenetic analysis made us sure that it was the larvae of *T*. *albipunctatus*. All these combinatory methods could be used to increase efficiency and precision of diagnosis.

Overall, through a morphological examination in combination with molecular biological methods, we accurately diagnosed the patient as having a *T*. *albipunctatus* infection.

## Ethics statement

The acquisition of samples was approved by the medical research ethics committee of Sun Yat-sen University. Informed consent was obtained from the subject, and the experiments were conducted according to the principles expressed in the Declaration of Helsinki. Written informed consent was obtained from the patient for publication of this Case Report and any accompanying images.

**Key learning points**

- *T*. *albipunctatus* is one of the medically important insects, especially in urban environments, that may potentially cause myiasis.
- This is the first case report about a Chinese middlescent woman infected with *T*. *albipunctatus* in Guangzhou, China.
- Combined with the results of morphological characteristics, PCR amplification, and phylogenetic analyses, we confirmed the larvae were *T*. *albipunctatus*.
- Clinician should pay attention to the infection of *T*. *albipunctatus*.
